# Dataset for optimized design parameters of three-phase induction motors with validation through machine learning

**DOI:** 10.1016/j.dib.2025.112274

**Published:** 2025-11-13

**Authors:** Upendra Kumar Potnuru, Srinivasa Kishore Teegala, Lakshmana Rao Kalabarige, Vidyabharati Ippili, M Raviraja Holla

**Affiliations:** a Department of Electrical and Electronics Engineering, GMR Institute of Technology, Rajam 532127, India; b Department of Computer Science and Engineering, GMR Institute of Technology, Rajam 532127, India; c CADFEM India Pvt. Ltd., Hyderabad 500082, India; d Manipal Institute of Technology, Manipal Academy of Higher Education, Manipal 576104, India

**Keywords:** Three phase induction motor, Design parameters, Design optimization, Performance characteristics, machine learning

## Abstract

Three-phase induction motors continue to dominate industrial and commercial sectors due to their high efficiency, robustness, and low maintenance requirements. This data article presents a curated dataset of optimized design parameters for three-phase induction motors covering output ratings from 0.5 kW to 100 kW. Motor parameters (stator/rotor dimensions, winding details, air-gap flux, copper/core losses, torque, slip, efficiency, power factor, currents, flux per pole, temperature rise, etc.) were computed by a Python based computational framework implementing standard electromechanical design equations. The original 200 design instances were scientifically expanded to 6000 to represent viable design alternatives. To demonstrate dataset reliability and practical utility, descriptive statistics and tree-based regressors (Decision Tree, Random Forest, Extra Trees) were applied on held out test sets and evaluated with MAE, RMSE, and R². The Extra Trees model yielded the lowest errors (e.g., MAE ≈ 7.31 W and RMSE ≈ 11.62 W for full-load losses; MAE ≈ 0.0073% and RMSE ≈ 0.0232% for efficiency) with R² ≳ 0.9996 and residuals concentrated near zero (≈0.0073–9.1536). These results confirm the internal consistency of the physics-driven dataset and its suitability for simulation, preliminary design studies, controller tuning, and predictive maintenance. However, the current dataset does not incorporate nonlinear magnetic effects, thermal constraints, or experimental validation, which will be addressed in future extended versions.

Specifications Table

**Table:** 

Subject	Electrical Engineering
Specific subject area	Electrical Machine Design
Type of data	Figures, tables, spread sheet file, Analysed, Processed
Data collection	The dataset in this study was generated using a Python-based computational model that incorporates fundamental electromechanical equations governing induction motor design. A Python script estimated parameters for output ratings from 0.5 kW to 100 kW, creating a structured dataset for design analysis. Statistical methods and machine learning were applied for validation, while data visualization revealed parameter trends and performance insights.
Data source location	GMR Institute of Technology, Rajam-532,127, Andhra Pradesh, India www.gmrit.edu.in
Data accessibility	Repository name: Three-Phase-Induction-Motor-Design-Data Data identification number: 10.5281/zenodo.16908384 Direct URL to data: https://github.com/KLakshmanarao/Three-Phase-Induction-Motor-Design-Data
Related research article	None

## 1 Value of Data

• The dataset offers optimized design parameters for three-phase induction motors, derived from standard equations and validated statistically. It supports motor selection, efficiency improvement, and performance analysis, serving as a reliable reference for engineers, researchers, and educators in advancing machine design, optimization, and sustainable applications.
• Researchers can reuse the dataset for motor modeling, simulation, and validation in MATLAB, ANSYS, and FEMM. It enables machine learning applications, predictive maintenance, and energy efficiency studies. The data supports teaching, benchmarking, and comparative research across motor ratings, fostering collaboration and innovation in electrical machine analysis and design.
• As a benchmark resource, the dataset validates theoretical models and simulations with standardized parameters. It improves reproducibility, accuracy, and comparison with experimental or manufacturer data, making it valuable for academic, industrial, and collaborative motor design research.
• The dataset captures variations in torque, slip, efficiency, and power factor, enabling trend analysis and energy-efficient motor design, while also providing accurate parameters for developing advanced controllers like PID, vector control, and DTC, thereby supporting VFD integration, torque–speed optimization, and innovations in industrial automation and smart manufacturing.
• By offering baseline motor characteristics, the dataset aids predictive maintenance and fault detection. It enables early identification of deviations, reduces downtime and costs, and supports development of reliable fault diagnosis algorithms for smarter, data-driven industrial applications.

## 2 Background

The motivation behind initiating this work stems from the need for a comprehensive, standardized dataset for 3-phase induction motors, bridging gaps in manufacturer data and aiding in motor selection across industries. Although extensive research exists on analytical modeling and optimization of induction motors, there is currently no publicly available dataset that consolidates key electrical, mechanical, and magnetic design parameters. By systematically generating these parameters, this work serves as a preliminary motor design tool, enabling engineers to estimate performance characteristics before engaging in complex finite element analysis (FEA) or prototype testing [[1], [2], [3]]. It enables AI-driven automation including motor behavioral predictions by machine learning, optimization of designs, and enhancement of predictive maintenance considered for Industry 4.0 applications. Academically, it contributes towards simulation and theoretical validation in a software environment such as MATLAB and ANSYS between the theoretical concepts and industrial application [[4], [5], [6]]. Furthermore, in line with Sustainable Development Goals (SDGs), this work acts as a driver for designing energy-efficient motors by facilitating power loss minimization, while also contributing to transitioning motors into the classes of IE3, IE4, and IE5. The generated dataset will also be vital for the implementation of digital twin technology for real-time monitoring, fault diagnosis, and predictive analytics in smart factories, making this work an initial step towards intelligent and data-driven motor design and operation, serving academia and industry, as well as moving towards a greener and efficient future [7,8].

## 3 Data Description

The dataset comprises optimized design parameters for three-phase induction motors with output power ratings ranging from 0.5 kW to 100 kW. The data is structured to deliver a complete understanding of motor performance characteristics, aiding in design, selection, and optimization. It consists of 200 rows and 97 columns, demonstrating a wide set of input, intermediate and output parameters. Each row corresponds to power output and columns correspond to various electrical and mechanical design parameters. The original dataset was further extended from 200 rows to 6000 rows to accommodate design alternatives in the dataset. The key parameters included in the dataset are categorized as follows:

### 3.1 Electrical design parameters

i. *Stator Voltage (V)*: The rated voltage of the motor, typically standardized (e.g., 230 V, 400 V, 690 V).
ii. *Stator and Rotor Current (A):* The current drawn by the stator and induced in the rotor, influencing power losses and thermal performance.
iii. *Power Factor (PF):* Indicates the phase difference between voltage and current, affecting efficiency and reactive power consumption.
iv. *Efficiency (%):* The ratio of output power to input power, which varies based on design choices like core material and winding configurations.
v. *Slip (%):* The relative difference between synchronous and rotor speed, a critical factor in torque production.

## 3.2 Mechanical design parameters

i. *Rotor Speed (RPM):* The actual operating speed of the motor, influenced by the slip and synchronous speed.
ii. *Torque (Nm):* The turning force generated by the motor, directly affecting load-handling capability.
iii. *Air Gap Length (mm):* The spacing between the stator and rotor, impacting magnetic flux and efficiency.
iv. *Frame Size and Core Dimensions (mm):* The physical dimensions of the motor, which determine thermal dissipation and mechanical strength.

### 3.3 Magnetic and thermal design considerations

i. *Flux Density (T):* The magnetic field strength in the stator and rotor core, optimized to prevent saturation while ensuring high efficiency.
ii. *Core and Copper Losses (W):* Thermal management is dependent upon various core losses such as hysteresis and eddy current losses and copper losses in winding resistance.
iii. *Temperature Rise (*°*C):* The insulation level is determined based on the motor temperature rise under full load conditions.

### 3.4 Computational and optimization insights

i. The dataset offers analysis of parameter variations, illustrating how motor performance will be affected based on design choices (mechanical - core dimensions, winding factors, and cooling mechanisms).
ii. It allows comparative analysis with respect to power ratings (electrical-efficiency trends, loss distribution, operational constraints, etc.)
iii. The structured dataset assists as a primary design tool for engineers, supporting in selection of motors based on the requirements for a specific application.

By including the design considerations mentioned above, the dataset connects theoretical modeling with real-world motor performance. It helps improve energy-efficient and high-performance induction motor designs.

## 4 Experimental Design, Materials and Methods

The typical design parameters of three phase induction motor are investigated using a computational experimental approach. The methodology involved development of programming code in Python to compute motor design parameters from fundamental electromechanical equations. Numerical computations, statistical analysis, and data visualization were performed in Python using libraries such as NumPy, Pandas, and Matplotlib, while MATLAB was utilized for cross-validation of key motor design parameters. The mathematical and theoretical foundation for the study was based on standard induction motor design equations, supported by IEEE and IEC standard references to ensure accuracy and compliance with established design practices [9].

### 4.1 Methodology

This study follows a structured data-driven approach:

**Step 1: Computation of Motor Parameters**

A Python script was developed to compute the following typical parameters for motor ratings from 0.5 kW to 100 kW:

• *Copper losses:* Stator copper loss, rotor bar loss, and end ring loss.
• *Efficiency:* Evaluated with respect to full-load losses and power output.
• *Flux Per Pole and Pole Pitch:* Magnetic circuit considerations for optimized performance.
• *Rotor Bar Current and Stator Phase Current:* Current distribution analysis for performance optimization.

**Step 2: Descriptive Statistical Analysis**

Descriptive statistics including mean, standard deviation, skewness, and kurtosis were employed to assess the consistency and distribution of parameters such as copper losses, efficiency, flux per pole, and rotor currents. These analyses revealed logical and physically meaningful trends (Table 1, Table 2, Table 3, Table 4, Table 5):

• Across all power ratings, the mean full load efficiency was found to be 68.2%, with a standard deviation of 4.7%, indicating moderate variation consistent with practical design expectations for medium-size induction motors.
• The mean copper losses increased linearly with output power, confirming the proportional relationship between current magnitude and I²R losses.
• Similarly, flux per pole showed a predictable rise with pole pitch, averaging 0.024 Wb, validating the correct implementation of magnetic circuit equations.
• The skewness and kurtosis values in Table 1, Table 2, Table 3, Table 4, Table 5 further confirmed data stability and realistic spread. Skewness values near zero (ranging from −0.18 to +0.17) suggest symmetric data distributions, indicating that the generated parameters are not biased toward extreme values.
• The consistently negative excess kurtosis values (≈ −1.2) indicate a platykurtic distribution, wider but flatter than a normal curve, reflecting controlled, non-peaked variations typical of computed engineering data without measurement noise. This ensures that the dataset represents physically consistent variations rather than random fluctuations.

**Table 1:** Descriptive statistics of the total copper losses with respect to copper loss in rotor bars.

Copper loss in bars	0-20%	20-40%	40-60%	60-80%	80-100%
*Mean*	*241.7619048*	*653.65625*	*1144.285714*	*1699.693878*	*2307.553571*
*Sum*	*5077*	*20917*	*48060*	*83285*	*129223*
*Minimum*	*37*	*430*	*883*	*1413*	*1995*
*Maximum*	*414*	*865*	*1399*	*1984*	*2619*
*Range*	*377*	*435*	*516*	*571*	*624*
*Variance*	*12177.70522*	*17039.03809*	*23484.10884*	*28125.47772*	*33592.56856*
*Standard Deviation*	*110.3526403*	*130.5336665*	*153.2452572*	*167.7065226*	*183.2827558*
*Median*	*251*	*656.5*	*1145*	*1700*	*2311*
*Excess Kurtosis*	*−1.098377817*	*−1.209639458*	*−1.204299313*	*−1.183150549*	*−1.191272461*
*Skewness*	*−0.188328172*	*−0.056463069*	*−0.031231626*	*−0.004739399*	*−0.010729536*
*Count*	*21*	*32*	*42*	*49*	*56*

**Table 2:** Descriptive statistics of the total copper losses with respect to copper loss in end rings.

Copper loss in End ring	0-20%	20-40%	40-60%	60-80%	80-100%
*Mean*	*416.7907*	*1009.902*	*1514.725*	*1981.59*	*2415.324*
*Sum*	*17922*	*41406*	*60589*	*77282*	*89367*
*Minimum*	*41*	*749*	*1279*	*1759*	*2210*
*Maximum*	*735*	*1264*	*1747*	*2199*	*2619*
*Range*	*694*	*515*	*468*	*440*	*409*
*Variance*	*39419*	*23427.89*	*19304.2*	*17192.75*	*14543.57*
*Standard Deviation*	*198.5422*	*153.0617*	*138.9396*	*131.1211*	*120.5967*
*Median*	*430*	*1013*	*1519*	*1984*	*2420*
*Excess Kurtosis*	*−1.11086*	*−1.20264*	*−1.21333*	*−1.20379*	*−1.19039*
*Skewness*	*−0.16474*	*−0.03216*	*−0.02285*	*−0.01887*	*−0.00178*
*Count*	*43*	*41*	*40*	*39*	*37*

**Table 3:** Descriptive statistics of the Efficiency with respect to full load losses.

Full Load Losses%	0-20%	20-40%	40-60%	60-80%	80-100%
*Mean*	*74.75236908*	*64.54208544*	*60.12700009*	*57.11335597*	*54.71487432*
*Sum*	*5307.418204*	*2581.683417*	*1924.064003*	*1656.287323*	*1532.016481*
*Minimum*	*67.55895309*	*61.96300815*	*58.49669754*	*55.8701777*	*53.67445549*
*Maximum*	*82.59854628*	*67.37847741*	*61.83790489*	*58.40038576*	*55.78849105*
*Range*	*15.03959319*	*5.415469257*	*3.341207346*	*2.530208056*	*2.114035565*
*Variance*	*21.96504538*	*2.565123667*	*0.989775166*	*0.570663483*	*0.401893813*
*Standard Deviation*	*4.686688103*	*1.601600345*	*0.994874447*	*0.755422718*	*0.633950955*
*Median*	*74.23776776*	*64.470746*	*60.10296022*	*57.10553379*	*54.70847291*
*Excess Kurtosis*	*−1.267725544*	*−1.183381613*	*−1.199774101*	*−1.201318129*	*−1.202264368*
*Skewness*	*0.177867639*	*0.108861959*	*0.050205298*	*0.056859944*	*0.037843818*
*Count*	*71*	*40*	*32*	*29*	*28*

**Table 4:** Descriptive statistics of Flux Per Pole with respect to Pole Pitch.

Pole Pitch	0-20%	20-40%	40-60%	60-80%	80-100%
*Mean*	*0.004114286*	*0.010777778*	*0.020184848*	*0.032877193*	*0.049011765*
*Sum*	*0.0288*	*0.194*	*0.6661*	*1.874*	*4.166*
*Minimum*	*0.0017*	*0.0067*	*0.0147*	*0.0255*	*0.04*
*Maximum*	*0.0062*	*0.0144*	*0.0252*	*0.0397*	*0.0575*
*Range*	*0.0045*	*0.0077*	*0.0105*	*0.0142*	*0.0175*
*Variance*	*2.20E-06*	*5.41E-06*	*9.59E-06*	*1.75E-05*	*2.60E-05*
*Standard Deviation*	*0.001482689*	*0.00232679*	*0.003097225*	*0.004186696*	*0.005098106*
*Median*	*0.0042*	*0.0109*	*0.0203*	*0.033*	*0.0492*
*Excess Kurtosis*	*−1.158553057*	*−1.172159424*	*−1.178408274*	*−1.195852144*	*−1.195372375*
*Skewness*	*−0.188220649*	*−0.136508493*	*−0.091123792*	*−0.080994108*	*−0.058176962*
*Count*	*7*	*18*	*33*	*57*	*85*

**Table 5:** Descriptive statistics of the rotor bar current with respect to stator current per phase.

Stator Current (Ph)	0-20%	20-40%	40-60%	60-80%	80-100%
*Mean*	*386.3*	*581.05*	*689.875*	*772.075*	*839.7*
*Sum*	*15452*	*23242*	*27595*	*30883*	*33588*
*Minimum*	*148*	*513*	*643*	*735*	*809*
*Maximum*	*508*	*641*	*733*	*808*	*870*
*Range*	*360*	*128*	*90*	*73*	*61*
*Variance*	*8787.01*	*1407.6975*	*708.259375*	*456.869375*	*327.51*
*Standard Deviation*	*93.73905269*	*37.51929504*	*26.6131429*	*21.37450292*	*18.09723736*
*Median*	*406.5*	*583.5*	*690.5*	*773.5*	*839.5*
*Excess Kurtosis*	*−0.328689782*	*−1.1670837*	*−1.197310283*	*−1.199454651*	*−1.184439344*
*Skewness*	*−0.731844952*	*−0.151177338*	*−0.101608837*	*−0.058028713*	*−0.033450839*
*Count*	*40*	*40*	*40*	*40*	*40*

These statistical indicators validate that the computed data follow expected electromechanical relationships, with no outliers or anomalies. The relationships observed such as the inverse dependence between full-load losses and efficiency, and the proportional relationship between stator and rotor currents, demonstrate strong internal coherence. Hence, the statistical measures not only summarize variability but also serve as diagnostic checks confirming that the dataset adheres to real-world motor performance behavior and is suitable for further modeling and machine learning analysis.

**Step 3: Variational Analysis**

Variations in key parameters were evaluated to determine performance dependencies (Fig. 1, Fig. 2, Fig. 3, Fig. 4, Fig. 5, Fig. 6, Fig. 7, Fig. 8, Fig. 9):

• Efficiency vs. Full-Load Losses and Power Output
• Stator Copper Loss vs. Stator Phase Currents and Resistance
• Rotor Copper Loss vs. Copper Loss in Rotor Bars and End Rings
• No-Load Current vs. Magnetizing Current and Loss Component of No-Load Current
• Flux Per Pole vs. Pole Pitch and Gross Iron Length
• Phase Resistance vs. Turns Per Phase and Mean Turn Length
• Phase magnetizing current vs turns per phase and magnetizing mmf per pole
• Rotor Bar Current vs. Turns Per Phase and Stator Current Per Phase
• Magnetizing MMF Per Pole vs. MMF Required for Rotor and Stator Core

**Fig. 1:**
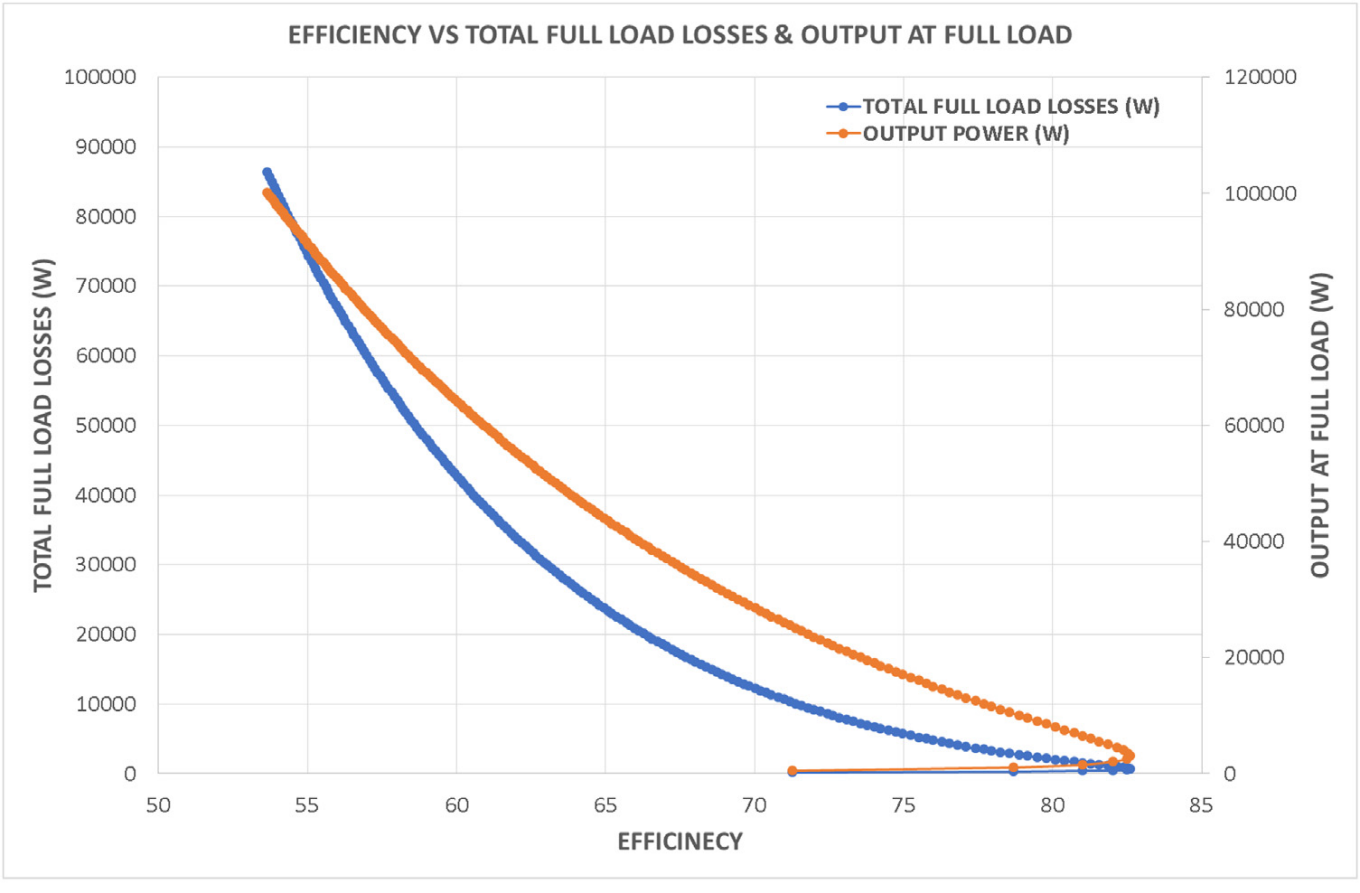
Variation of efficiency with respect to full load losses and power output.

**Fig. 2:**
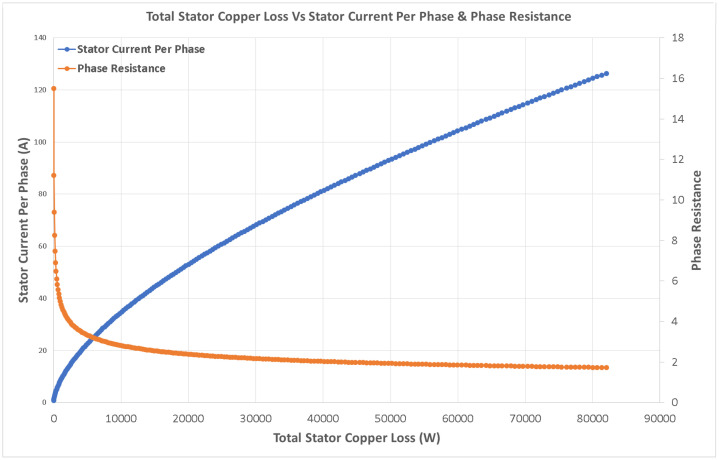
Variation of stator copper loss with respect to stator phase currents and resistance.

**Fig. 3:**
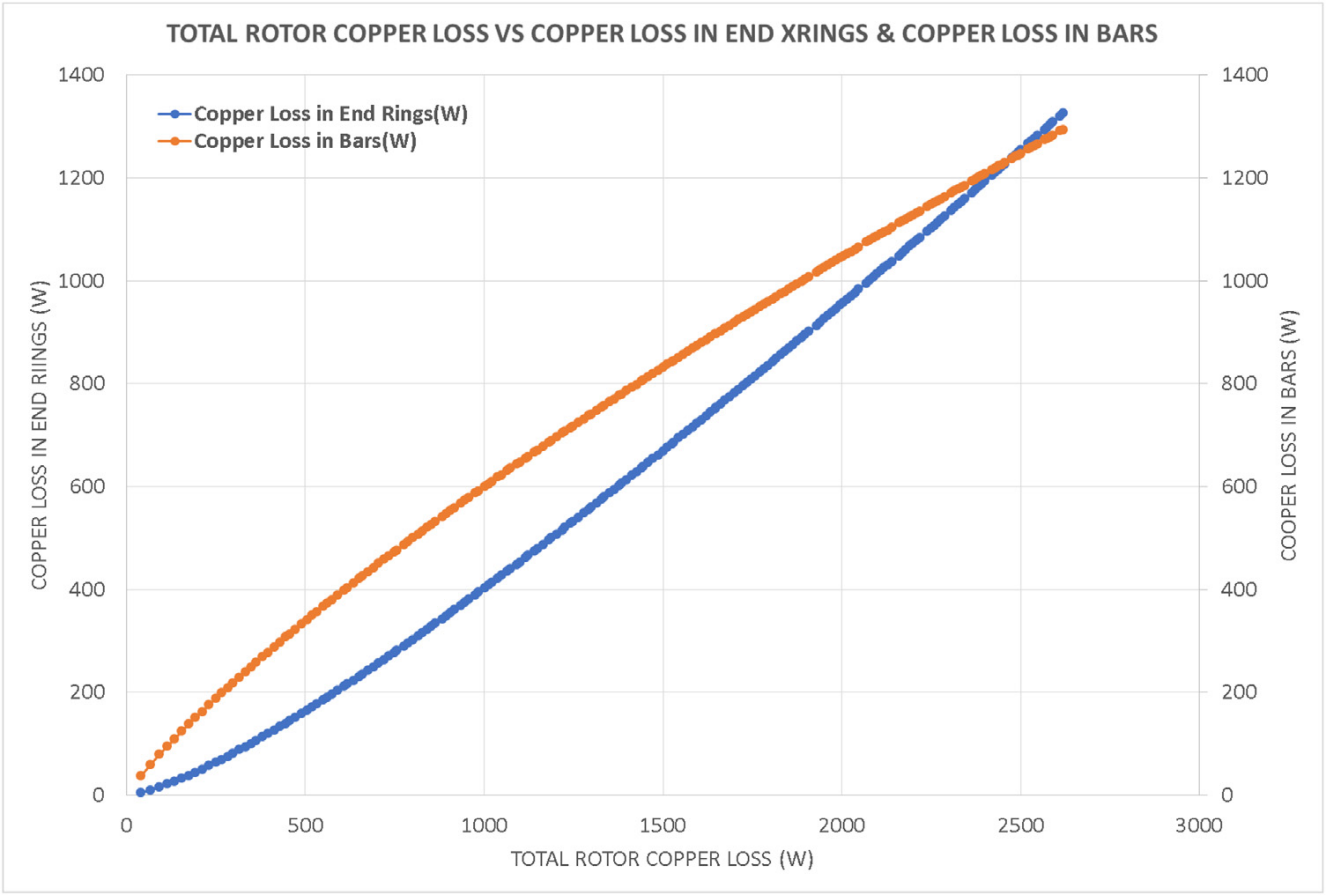
Variation of rotor copper loss with respect to copper loss in rotor bars and end rings.

**Fig. 4:**
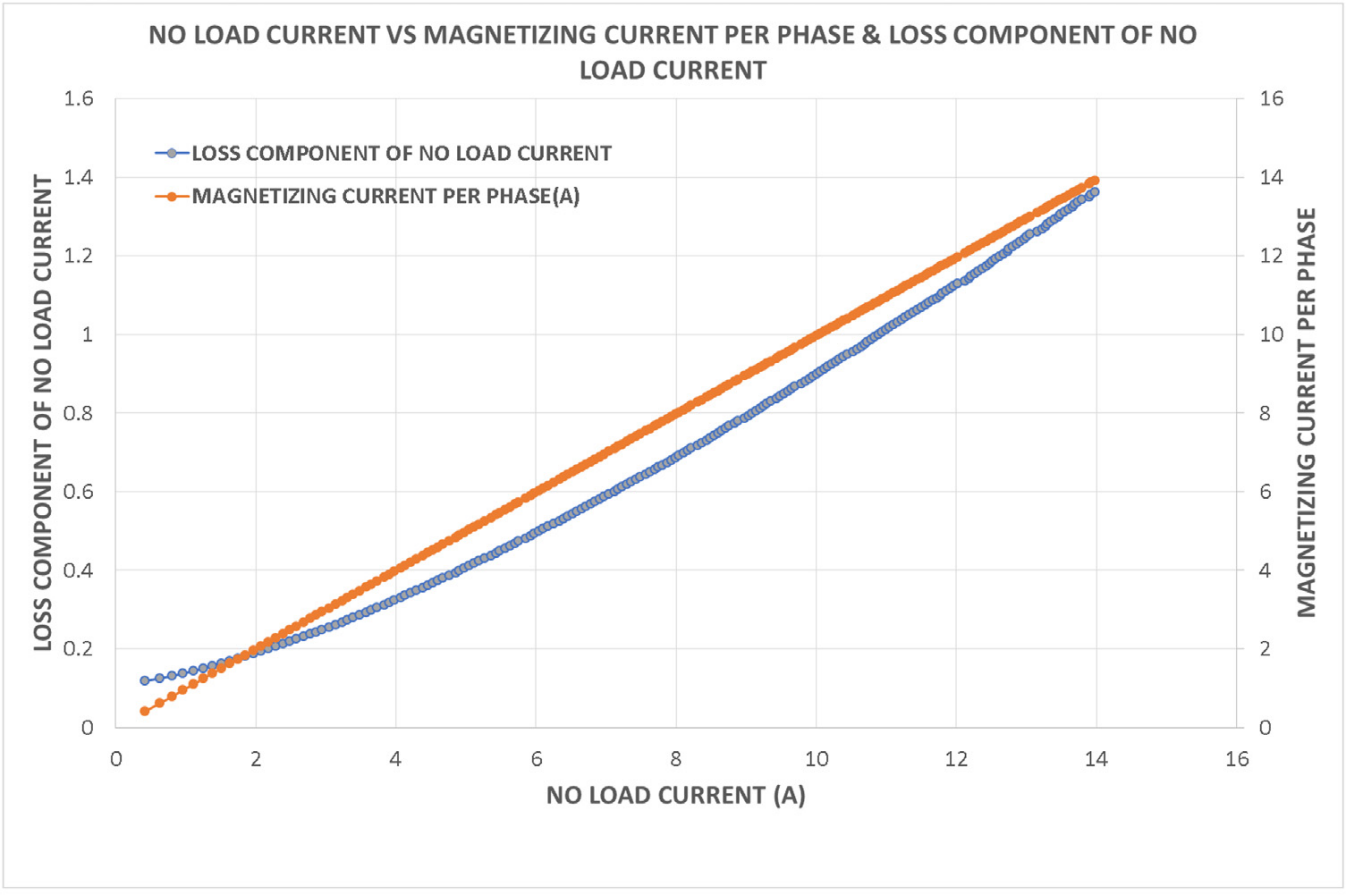
Variation of no load current vs magnetizing current and loss component of no load current.

**Fig. 5:**
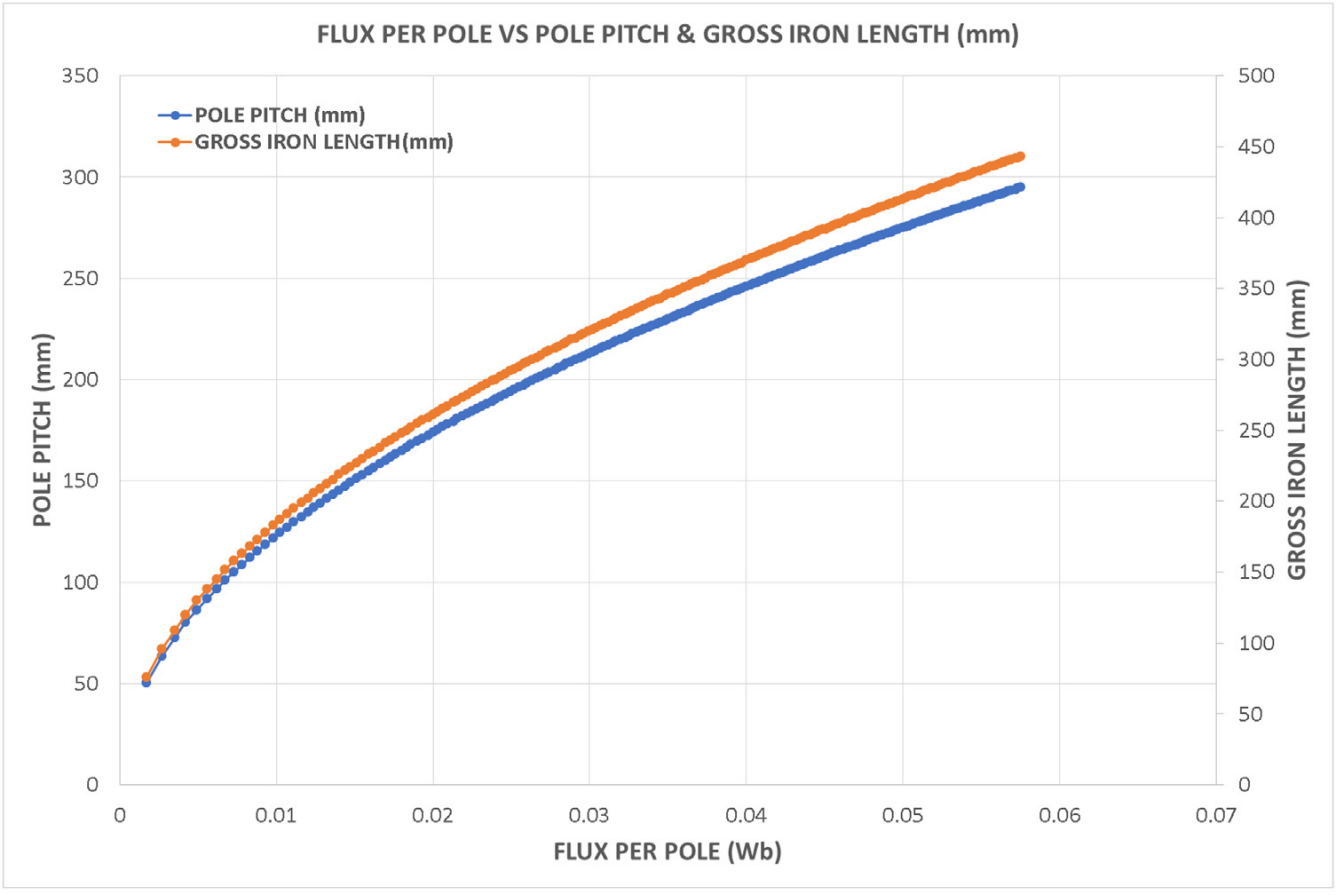
Variation of flux per pole vs pole pitch and gross iron length.

**Fig. 6:**
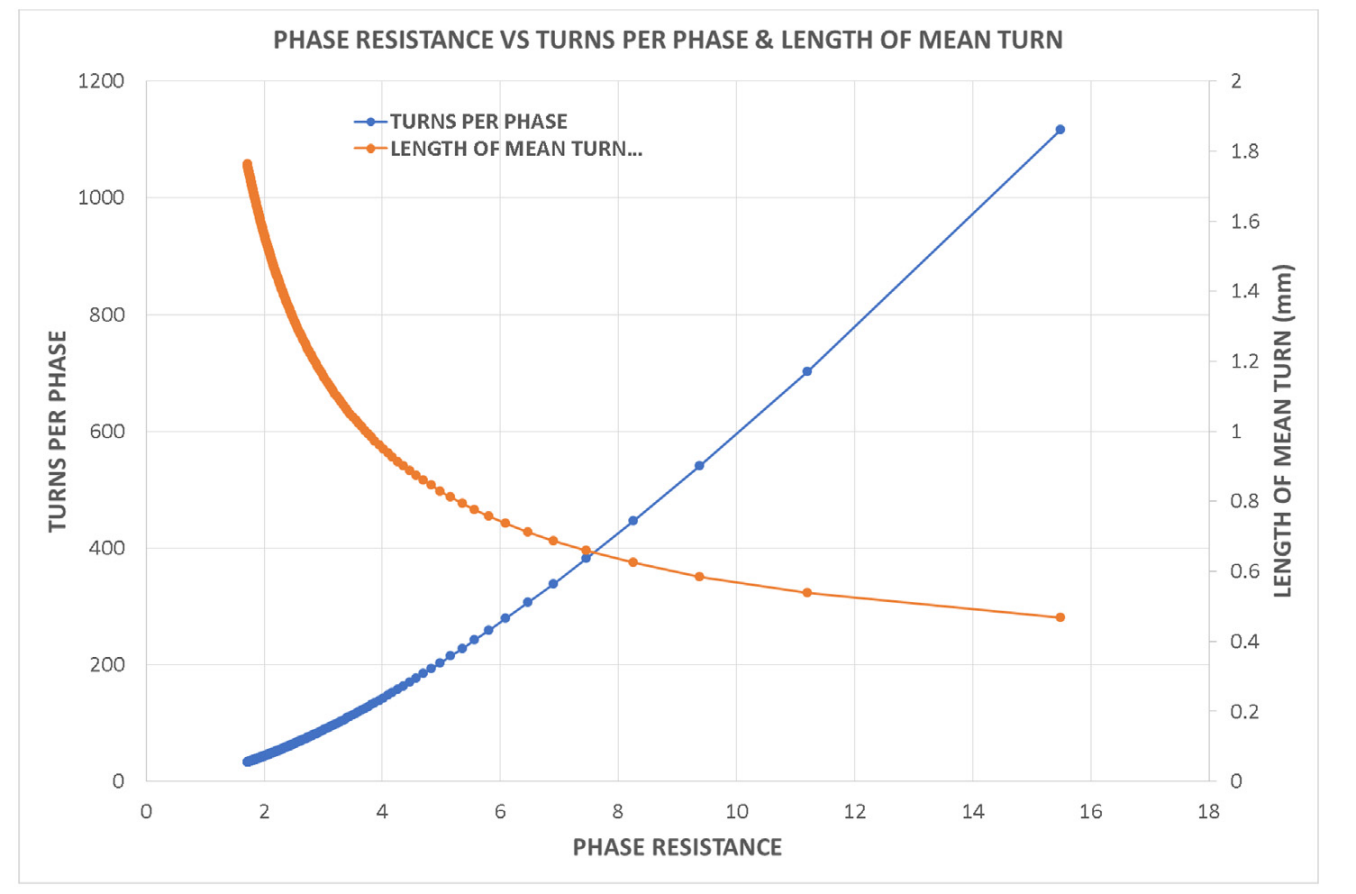
Variation of phase resistance vs turns per phase and mean turn length.

**Fig. 7:**
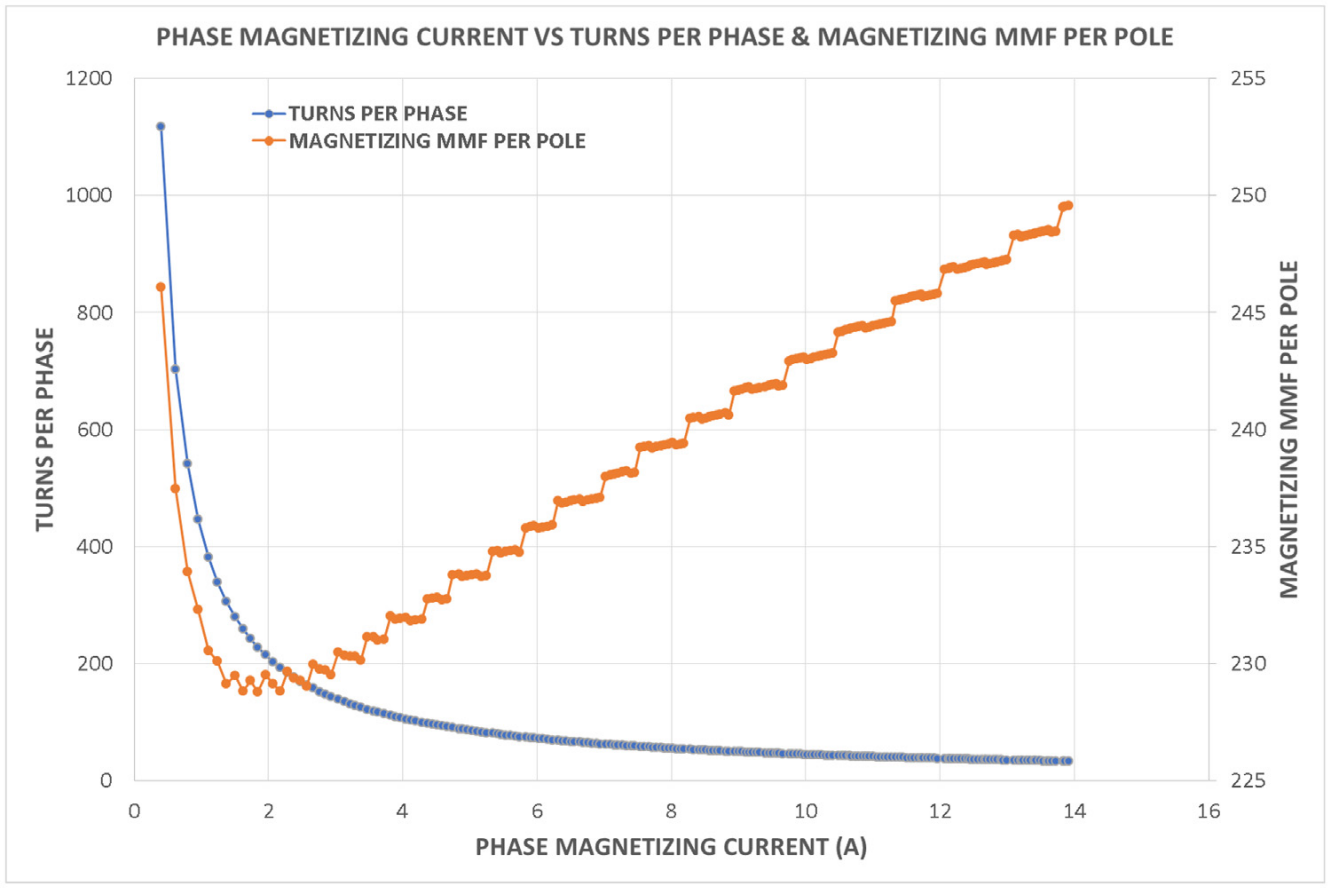
Variation of phase magnetizing current vs turns per phase and magnetizing mmf per pole.

**Fig. 8:**
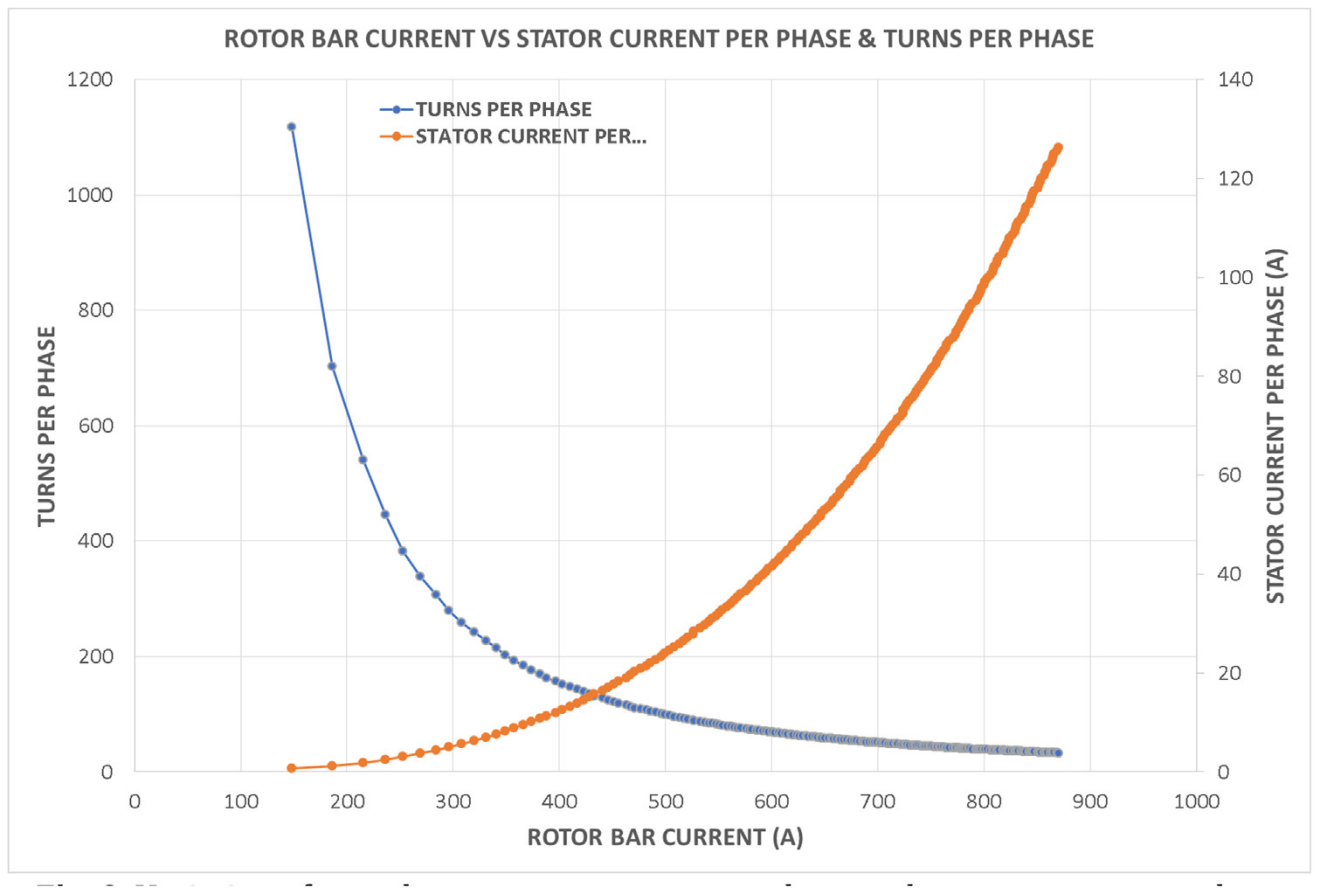
Variation of rotor bar current vs turns per phase and stator current per phase.

**Fig. 9:**
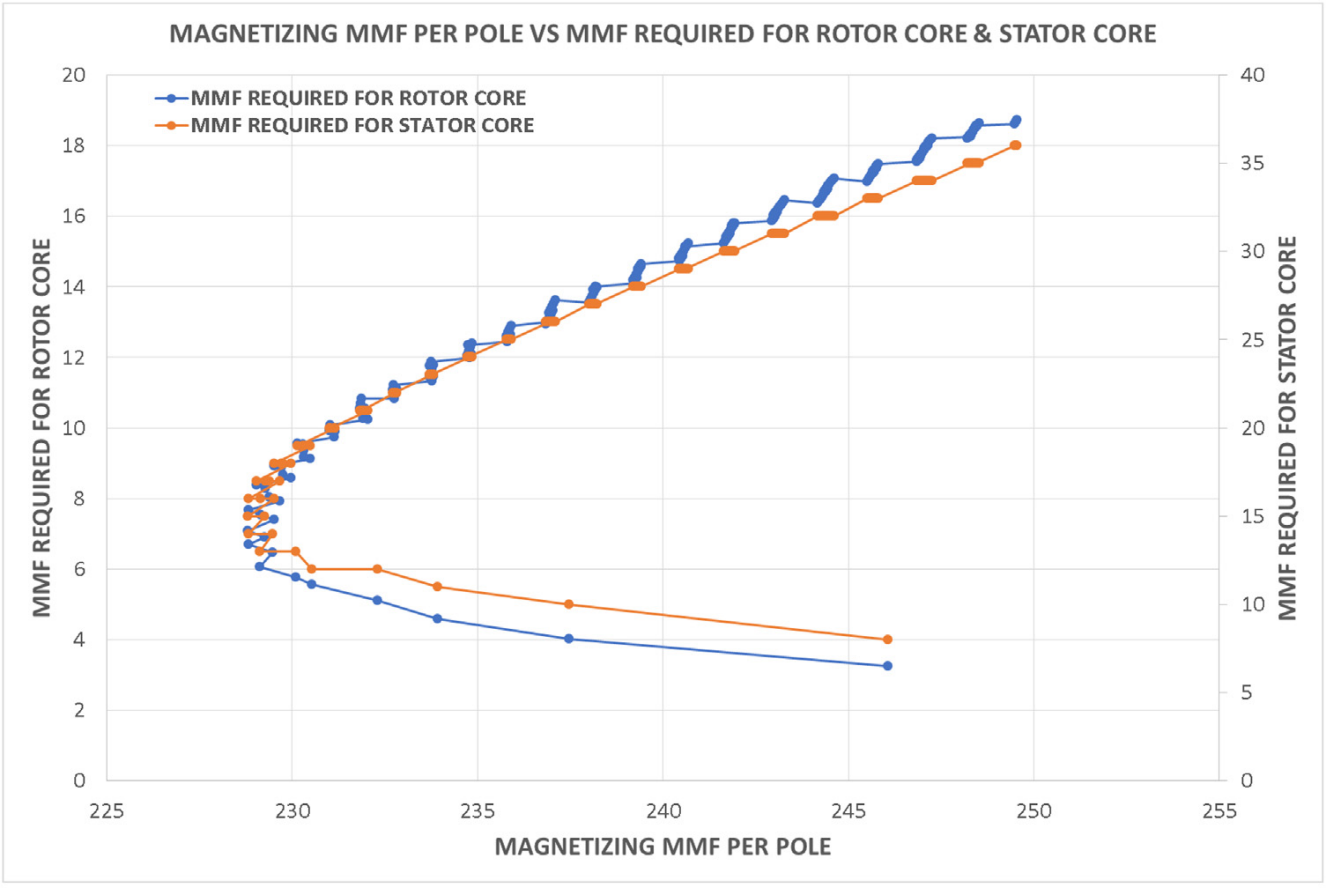
Variation of magnetizing mmf per pole vs mmf required for rotor and stator core.

**Step 4: Validation and Interpretation**

To ensure that the machine learning results reflect genuine predictive capability rather than overfitting or data leakage, a rigorous validation protocol was implemented. The dataset was divided using a 70:30 train–test split, and all models were trained only on the training portion while the test set remained unseen during training. Additionally, a five-fold cross validation procedure was employed to obtain the learning curves. The input features were pre-processed and normalized to prevent dominance of higher magnitude variables. No overlapping or duplicate entries existed between training and testing subsets, eliminating data leakage. A series of tree-based regression models, specifically Decision Tree Regressor (DTR), Random Forest Regressor (RFR), and Extra Trees Regressor (ETR) were compared based on Mean Absolute Error (MAE), Root Mean Squared Error (RMSE), and R²-score for each target variable.

**Fig. 10:**
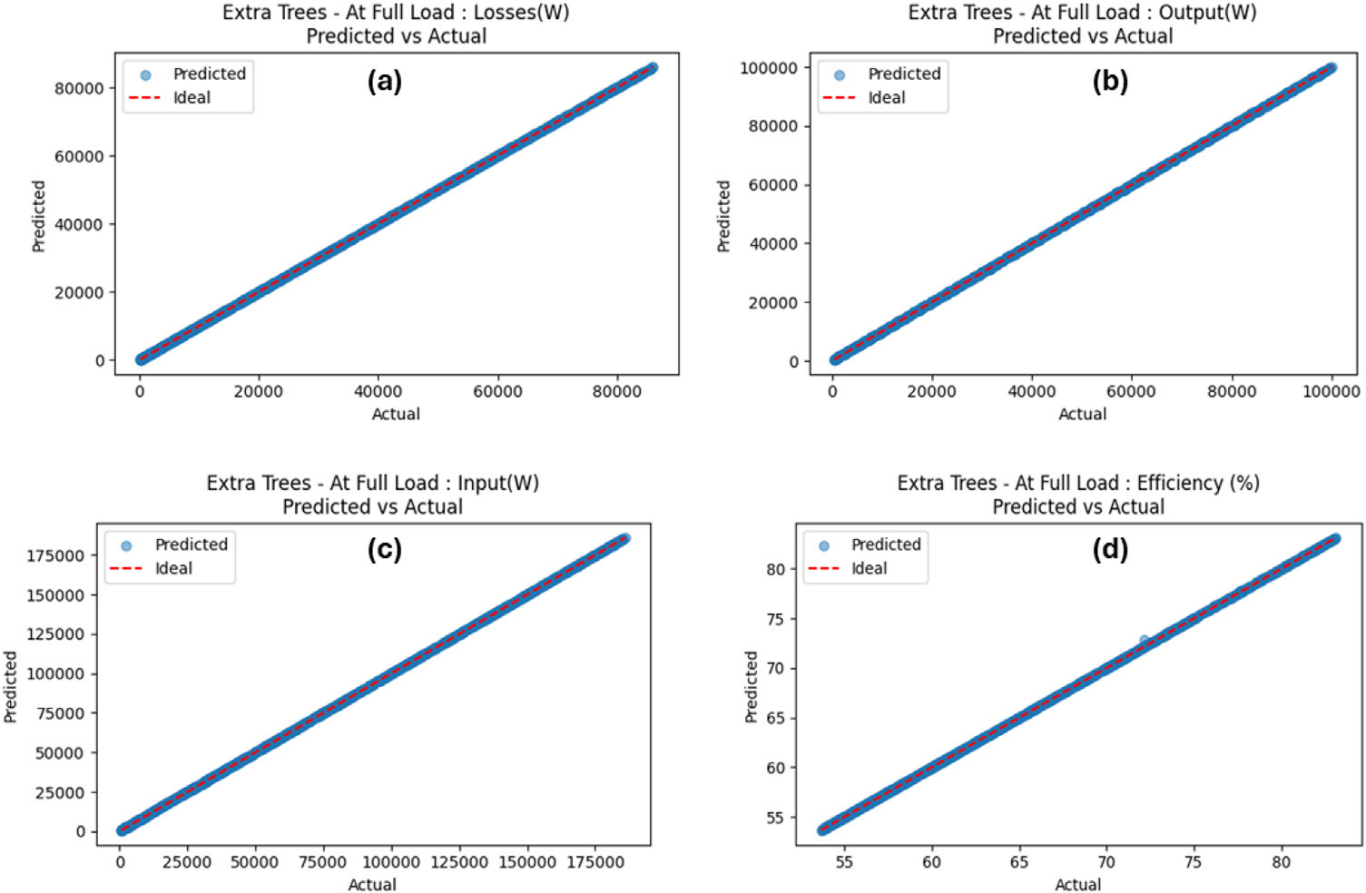
Comparison of ETR model for predicted and actual parameter values.

**Fig. 11:**
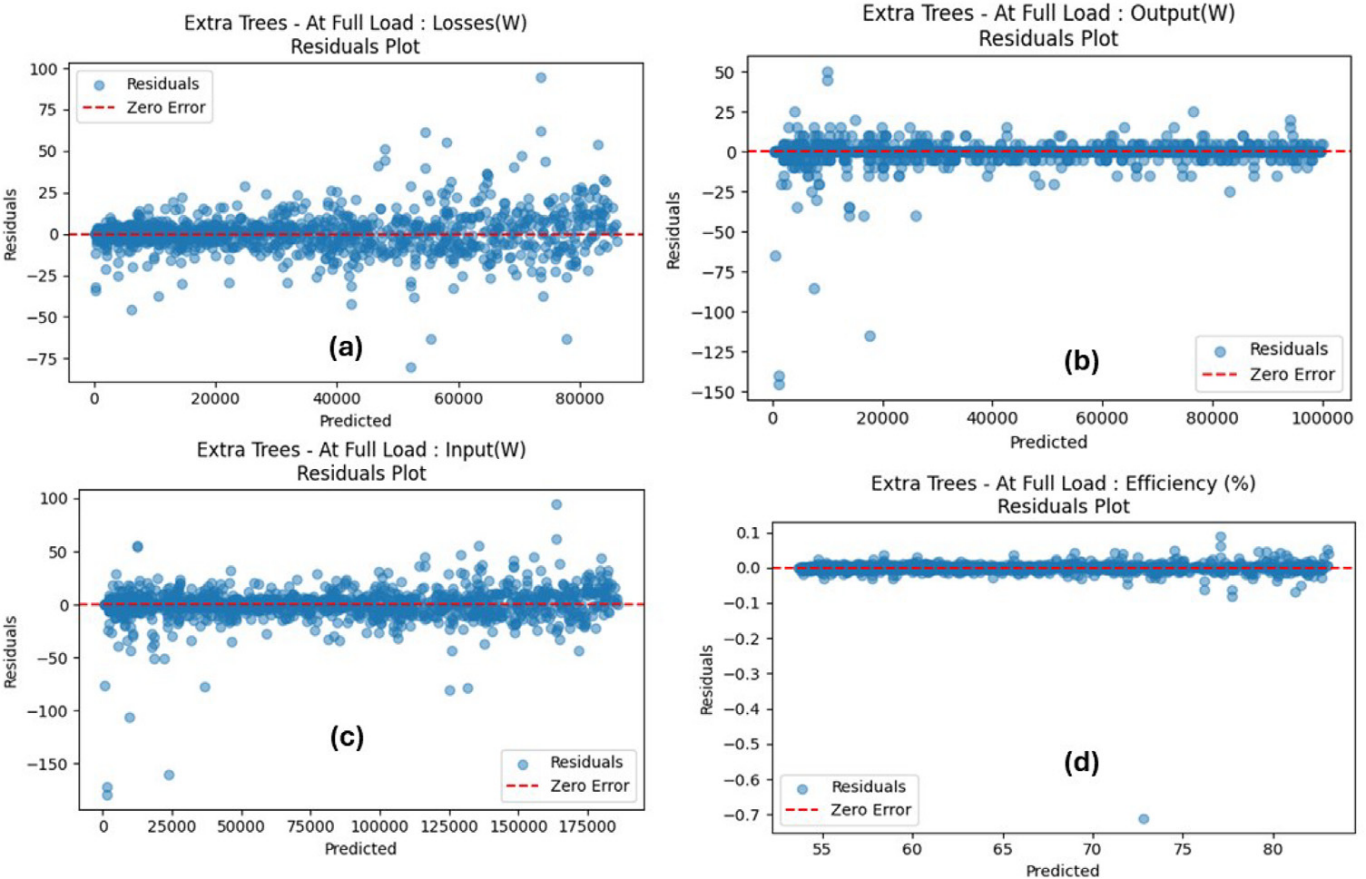
Comparison of ETR model for Residuals.

According to Table 6, the findings show that all three models considered performed well, since the predicted values were very close to the actual values. The R²-score was especially high across all target features, achieving 1.0000 for most of the cases, while the least R²-score was 0.9996 for efficiency prediction using the DTR model. Although the R² values approached unity (0.9996–1.0000), this outcome is expected due to the deterministic nature of the dataset. The data was generated from well-defined electromechanical equations without measurement noise, meaning that each target variable is a direct function of the input parameters. Hence, models trained on such physics-derived data are expected to reproduce results with minimal residuals. With the ETR producing the lowest values of MAE and RMSE across all target features considered, this model outperformed all other models. Taking into consideration the strongly correlated predicted versus actual values (Fig. 10) for the ETR model, the interpretation appears to be that outcomes predicted strongly coincide with ideal (actual) values. Further scrutinization of recorded differences between the actual and predicted values for the ETR model is presented in Fig. 11, with the residual values existing in an interval range from 0.0073 to 9.1536. The plots show a consistent distribution of residuals around the zero-error line, confirming the model's high predictive accuracy and robustness.

**Table 6:** Performance of the tree-based models.

Outcome	Model	MAE	RMSE	R^2^
*At Full Load: Losses(W)*	*DTR*	*25.6554*	*46.7011*	*1.0000*
	*RFR*	*12.2728*	*17.8797*	*1.0000*
	*ETR*	*7.3062*	*11.6153*	*1.0000*
*At Full Load: Output(W)*	*DTR*	*6.6667*	*57.7350*	*1.0000*
	*RFR*	*12.3625*	*21.9597*	*1.0000*
	*ETR*	*2.9708*	*9.3307*	*1.0000*
*At Full Load: Input(W)*	*DTR*	*32.3220*	*96.2699*	*1.0000*
	*RFR*	*22.3149*	*33.6927*	*1.0000*
	*ETR*	*9.1536*	*15.9870*	*1.0000*
*At Full Load: Efficiency (%)*	*DTR*	*0.0284*	*0.1717*	*0.9996*
	*RFR*	*0.0119*	*0.0342*	*1.0000*
	*ETR*	*0.0073*	*0.0232*	*1.0000*

**Fig. 12:**
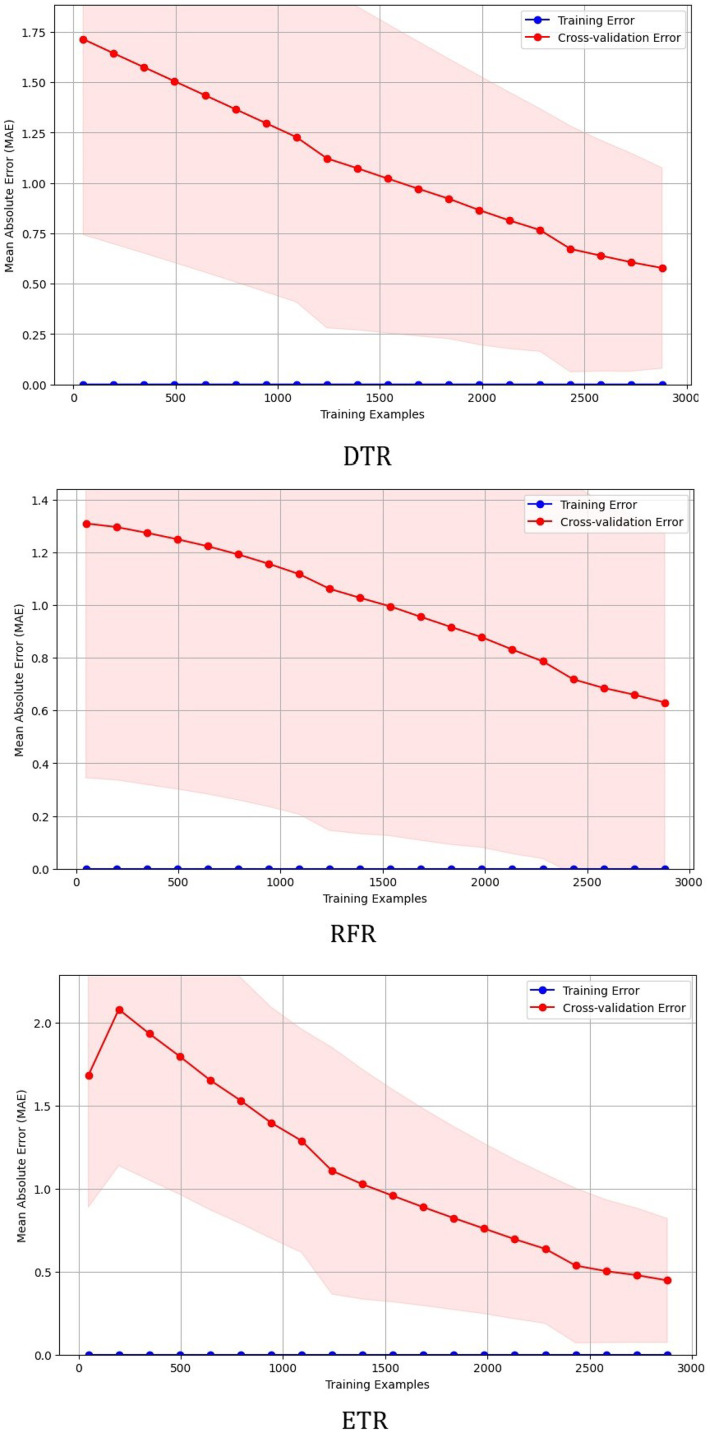
Learning curves for tree-based models.

Learning curves for the DTR, RFR, and ETR models were plotted by varying the training data and are illustrated in Fig. 12. As shown in Fig. 12, the training error (blue curve) remains nearly constant and close to zero across all models, indicating that the algorithms successfully fit the underlying relationships within the dataset. The cross-validation error (red curve) decreases steadily with an increase in training size, showing that each model improves generalization as more data is provided. The convergence of the two curves at low error values confirms that the models exhibit low bias and low variance, with no signs of overfitting or data leakage. The smooth decline of validation error further supports the deterministic nature of the dataset, which is derived from exact electromechanical equations rather than noisy experimental measurements. Among the three models, the ETR demonstrates the most stable and minimal cross-validation error, reaffirming its superior predictive accuracy and robustness for modeling induction motor performance parameters. These findings highlight the potential of applying machine learning models to the synthesized dataset for accurate and efficient prediction of induction motor performance characteristics, which can serve as a valuable tool for design, optimization, and predictive maintenance in industrial applications.

## Limitations

The following areas of machine design still pose a challenge which this dataset cannot deal with.

• First, material property effects such as magnetic saturation, hysteresis, and temperature-dependent resistivity were not incorporated. The dataset assumes constant material behavior, which may cause minor overestimation of efficiency and underestimation of losses at higher loads or temperatures. Incorporating manufacturer-specific material data or nonlinear B–H characteristics in future versions would improve physical accuracy.
• Second, thermal and mechanical constraints were not explicitly modelled. The present framework does not account for heat dissipation, cooling efficiency, or mechanical stresses, which could influence copper losses, insulation class selection, and lifetime predictions. Future extensions integrating finite element analysis (FEA) or computational fluid dynamics (CFD) can help couple electromagnetic and thermal models for more realistic outcomes.
• Third, manufacturing and geometric feasibility including slot geometry, tolerances, and winding configurations was simplified for computational generalization. This abstraction limits the dataset’s use for direct prototyping but retains its value for conceptual and comparative design.
• Finally, the numerical framework assumes linear magnetic behavior and ideal sinusoidal supply, excluding harmonic distortion and stray-load losses. As a result, efficiency and torque predictions may differ slightly from real-world machine performance. These nonlinearities can be addressed in future datasets through FEA-based magnetic modeling or experimental validation.

Overall, these limitations highlight that while the dataset provides a robust foundation for design optimization, selection, and performance prediction, it should be complemented with experimental, FEA, or manufacturer data for high-fidelity motor design and industrial deployment.

## Ethics Statement

The authors have read and follow the ethical requirements for publication in Data in Brief and confirming that the current work does not involve human subjects, animal experiments, or any data collected from social media platforms.

## Credit Author Statement

**Upendra Kumar Potnuru:** Conceptualization, Methodology, Software; **Teegala Srinivasa Kishore:** Writing - Original Draft, Formal analysis, Validation; **Lakshmana Rao Kalabarige:** Writing - Review and Editing, Data Curation, Visualization; **Vidyabharati Ippili:** Supervision; **Raviraj Holla M:** Review and Editing, Supervision.

## Data Availability

GIT HubThree-Phase-Induction-Motor-Design-Data (Original data).

## Appendix Supplementary materials

Image, application 1
