# Supplementary material for: Dataset for optimized design parameters of three-phase induction motors with validation through machine learning
Source: Data Brief. 2025 Nov 13;63:112274. doi: 10.1016/j.dib.2025.112274 (PMC12686921; doi:10.1016/j.dib.2025.112274)
Supplement: Supplementary file 1 [file mmc1.pdf]

# Induction Motor Design Equations

- Reference: A Course In Electrical Machine Design by A.K. Sawhney , Dhanpat Rai & Co. (P) Limited, ISBN-10 : 8177001019, ISBN-13 : 978-8177001013, $6^{th}$ Edition (Reprint), 2022.

## Nomenclature

| | |
|---|---|
| P = Number of poles | $l_a$ = Length of air gap |
| f = Frequency | $D_r$ = Diameter of rotor |
| $n_s$= Synchronous speed | $S_r$ = Number of rotor slots |
| kW = Full load output | $q_r$ = Slots per pole per phase |
| Q = kVA input | $z_{sr}$ = Conductor per slot |
| $E_s$ = Phase voltage | $k_{wr}$= Rotor winding factor |
| $I_s$ = Stator current per phase | $y_{sr}$ = Slot pitch |
| $B_{av}$ = Specific magnetic loading | $I_b$ = Rotor bar current |
| $\varphi_m$ = Flux per pole | $a_b$ = Area |
| D = Stator core | $L_b$ = Length |
| $L_s$ = Gross iron length | $\delta_b$ = Current density |
| ac = Specific electric loading | $r_b$ = Resistance of each bar |
| $C_0$ = Output coefficient | $I_e$ = End ring current |
| $K_w$ = Winding constant | $a_e$ = Area |
| $n_d$ = Ducts | $D_e$ = Mean diameter |
| $L_i$ = Net iron length | $\delta_e$ = Current density |
| τ = Pole pitch | $r_e$= Resistance of each ring |
| $T_s$ = Turns per phase | $r_r$ = Resistance of rotor |
| $S_s$ = Number of slots | $d_{cr}$ = Depth of rotor core |
| $q_s$ = Slots per pole per phase | $AT_{60}$= Magnetizing mmf per pole |
| $C_s$ = Coil span | $I_m$= Phase magnetizing current |
| $K_d$ = Distribution factor | $X_m$ = Magnetizing reactance |
| $K_p$ = Pitch factor | $I_l$ = Loss component |
| $K_{ws}$ = Stator winding factor | $I_{sl}$= No load current [line] |
| $y_{ss}$ = Stator slot pitch | $I_0$ = No load current [phase] |
| $S_c$ = Total stator conductors | $\cos\phi_0$ = No load power factor |
| $z_{ss}$ = Conductor per slot | $x_s$ = Slot leakage reactance |
| d = Conductor bare diameter | $X_0$= Overhang leakage reactance |
| $a_s$ = Conductor area | $X_z$ = Zigzag leakage reactance |
| $\delta_s$ = Current density | $X_t$ = Total leakage reactance |
| $L_{mts}$ = Length of mean turn | $R_t$ = Total resistance |
| $r_s$ = Phase resistance | $Z_s$ = Short circuit impedance |
| $d_{cs}$ = Depth of stator core | $I_{sc}$= Phase short circuit current |
| $D_c$ = Outer diameter of stator laminations | $\cos\phi_{sc}$ = Short circuit power factor |
| $d_{ss}$ = Depth of the stator slot | $\theta_m$ = Temperature raise |

# Formulae

1. No. of poles (p) = $\left(\frac{2f}{n_s}\right)$

2. Synchronous speed ($n_s$) = $\left[\frac{\text{synchronous r.p.m}}{60}\right]$ r.p.s

3. kVA input (Q) = $\left[\frac{\text{kW}}{\text{power factor}\times\text{efficiency}}\right]$

4. Stator current per phase ( $I_s$) =$\left[\frac{\text{kW}}{3\times E_s\times\text{efficiency}\times\text{power factor}}\right]$ A

5. Full load line current = $(\sqrt{3} \times I_s)$ A

6. Specific magnetic loading ($B_{av}$) = $\left[\frac{\varphi_m\times p}{\pi\times D\times L_s}\right]$ Wb/m$^2$

7. Output coefficient ($C_0$) =$(11 \times K_w \times B_{av} \times ac \times 10^{-3})$

8. $D^2L = \frac{Q}{(C_0\times n_s)}$

9. Stator core (D) =$\left(\frac{p}{\pi} \times \sqrt{0.18}\right)$m

10. Net iron length ($L_i$) =$(0.9\times L_s)$ mm

11. Pole pitch ($\tau$) =$\sqrt{0.18 \times L_s}$

12. Flux per pole ($\phi_m$) = $(B_{av} \times \tau \times L_s)$ Wb

13. Turns per phase ($T_s$) =$\left[\frac{E_s}{4.44\times f\times\phi_m\times K_w}\right]$

14. No. of slots ($S_s$) =$(3\times p \times q_s)$

15. Coil span ($C_s$) =$\left[\frac{S_s}{p}\right]$

16. Distribution factor ($K_d$) =$\left[\frac{\sin\frac{q_s\times\theta}{2}}{\sin\frac{\theta}{2}}\right]$

17. Pitch factor ($K_p$) =$\left[\cos\frac{\theta}{2}\right]$

18. Stator winding factor ($K_{ws}$) =$(K_d \times K_p)$

19. Slot pitch ($y_{ss}$) =$\left(\pi \times D \times \frac{10^3}{S_s}\right)$

20. Total stator conductors ($S_c$) =$(6\times T_s)$

21. Conductor per slot ($z_{ss}$) =$\left[\frac{S_c}{S_s}\right]$

22. Conductor Area ($a_s$)=$\left[\frac{\pi}{4} \times d^2\right]$ mm$^2$

23. Current density ($\delta_s$) =$\left[\frac{I_s}{a_s}\right]$ A/mm$^2$

24. Length of mean turn ($L_{mts}$) = $[(2 \times L) + (2.3 \times \tau) + S_s]$ mm

25. Copper loss at full load $=3 \times I_s^2 \times r_s$

26. Depth of stator core $(d_{cs}) = \left[\frac{\text{Area of stator core}}{L_i}\right]$

27. Outer diameter of stator laminations $(D_c) = (D + 2\,d_{ss} + 2d_{cs})$

28. Length of air gap $(l_a) = \left(0.2 + 2\sqrt{D \times L}\right)$

29. Diameter of rotor $(D_r) = (D - 2 \times l_a)$

30. No. of rotor slots $(S_r) = (S_s - q_s)$

31. Slot pitch $(y_{sr}) = \pi \times \left(\frac{Dr}{s_r}\right)$

32. Rotor bar current $(I_b) = \frac{2 \times m_S \times k_{ws} \times T_Y}{S_r} \times I_S \cos\phi$

33. Rotor bar Area $(a_b) = I_b / \delta_b$

34. Resistance of each bar $(r_b) = \frac{\rho \times L_b}{a_b}$

35. Copper loss in bars $= S_r \times I_b^2 \times r_b$

36. End ring current $(I_e) = \frac{S_r \times I_b}{\pi \times p}$

37. End ring Area $(a_e) = I_e / \delta_e$

38. Resistance of each ring $(r_e) = \frac{\rho \pi D_e}{a_e}$

39. Copper loss in bars $=2 \times I_e^2 \times r_e$

40. Total rotor copper loss $=(S_r \times I_b^2 \times r_b) + (2 \times I_e^2 \times r_e)$

41. Resistance of rotor $(r_r) = \left[\rho \times \left(\frac{T_r \times L_{mtr}}{a_n}\right)\right]\Omega$

42. Depth of rotor core $(d_{cr}) = \left[\frac{\varphi_m}{2 \times B_{cr} \times L_i}\right]$

43. Phase magnetizing current $(I_m) = \frac{0.427 \times p \times AT_{60}}{k_{ws} \times T_s}$

44. Magnetizing reactance $(X_m) = E_s / I_m$

45. Core loss $=[2 \times (\text{loss per kg} \times \text{weigth of stator teeth} + \text{iron loss in core})]$

46. Friction and windage loss $= \left[\frac{1.5}{100} \times kW\right]$

47. No load loss $=(\text{iron loss} + \text{friction and windage loss})$

48. Loss component $(I_l) = \left[\frac{\text{total no load loss}}{3 \times \text{voltage per phase}}\right]$

49. No load current [line] $(I_{sl}) = \left(\sqrt{3} \times I_0\right)$

50. No load current [phase] $(I_0) = \sqrt{(I_m^2) + (I_l^2)}$

51. No load power factor $(\cos\phi_0) = \frac{I_l}{I_0}$

52. Slot leakage reactance $(x_s) = \left[8 \times \pi \times f \times T_{ph}^2 \times L \times \left(\frac{\tau_s}{p \times q}\right)\right]$

53. Overhang leakage reactance $(X_0) = \left[8 \times \pi \times f \times T_s^2 \times \left(\frac{L_0 \times \tau_0}{p \times q_s}\right)\right]$

54. Zigzag leakage reactance $(X_z) = \left[\frac{5}{6} \times \frac{X_m}{M_s^2} \times \left(\frac{1}{q_s^2} + \frac{1}{q_r^2}\right)\right]$

55. Total leakage reactance $(X_t) = (X_s + X_z + X_0 + X_h)$

56. Total resistance $(R_t) = (\text{copper loss at full load} + r_r)$

57. Short circuit impedance $(Z_s) = \sqrt{(X_s^2) + (R_s^2)}$

58. Phase short circuit current $(I_{sc}) = \frac{X_m}{Z_s}$

59. Line short circuit current $= \left(\sqrt{3} \times I_{sc}\right)$

60. Short circuit p.f $(\cos \phi_{sc}) = \frac{R_s}{Z_s}$

61. Full load Efficiency $= \left(\frac{\text{output}}{\text{output+losses}}\right)$

62. Full load Power factor $= \frac{p}{s}$

63. Full load Slip $= \left(\frac{n_s - n_r}{n_s}\right) \times 100$

64. Temperature raise$(\theta_m) = \left[\frac{\text{total loss to be dissipated}}{\text{loss dissipated /°C rise of temperature}}\right]$

# Assumptions

1. Specific electric loading (AC) = 21000 A/m
2. Ducts ($n_d$) =Nil
3. Type of laminations =0.5 mm
4. Gross iron length ($L_s$) =0.125 m
5. Type of winding =Single layer mesh
6. Connection =Delta
7. Phase voltage ($E_s$) =400 V
8. Slots per pole =6
9. Slots per pole per phase ($q_s$) =2
10. Conductor: Bare diameter (d)=0.95 mm; Insulated diameter = 1.041 mm
11. Phase resistance at 75°C ($r_s$) =8.37Ω
12. Type of winding =Squirrel cage
13. Slots per pole per phase ($q_r$) =1.835
14. Conductor per slot ($z_{sr}$) =1
15. Rotor winding factor ($k_{wr}$) =1
16. Rotor bar: Cross-section =7×6.5mm; Length ($L_b$) =165mm; Current density ($\delta_b$) =5.47 A / $mm^2$
17. End ring: Cross-section =$10 \times 8$ $mm^2$; Mean diameter ($D_e$) =75.8mm; Current density ($\delta_e$) = 5 A / $mm^2$
18. Magnetizing mmf per pole ($AT_{60}$)=231 A
